# Simultaneous spectrophotometric determination of finasteride and tadalafil in recently FDA approved Entadfi™ capsules

**DOI:** 10.1186/s13065-022-00850-w

**Published:** 2022-07-29

**Authors:** Ahmed H. Abdelazim, Sherif Ramzy

**Affiliations:** Pharmaceutical Analytical Chemistry Department, Faculty of Pharmacy, Al-Azhar University, Nasr City, Cairo, 11751 Egypt

**Keywords:** Entadfi™, Finasteride, Tadalafil, Derivative spectroscopy.

## Abstract

Entadfi™ is a recently FDA approved pharmaceutical combination capsule of finasteride and tadalafil. It was prescribed for the treatment of urinary tract disorders caused by benign prostatic hyperplasia in men. This paper introduced the first spectrophotometric methods for simultaneous determination of finasteride and tadalafil in the pure form and in the pharmaceutical capsules. UV absorption spectra of finasteride and tadalafil exhibited overlap hindered the direct simultaneous determination of the cited drugs. The UV absorption spectra of finasteride and tadalafil were transformed to the second order derivative. Finasteride could be determined selectively at 230.80 nm without interference from tadalafil. Moreover, tadalafil could be determined selectively at 292 nm without interference from finasteride. The ratio spectra of the studied drugs were derived and the derived ratio spectra of each drug were transformed to the first order derivative. Finasteride could be determined selectively at 218.80 nm without interference from tadalafil. Moreover, tadalafil could be determined selectively at 289.60 nm without interference from finasteride. The methods showed linearity with an excellent correlation coefficient in the concentration range of 10–140 µg/mL for finasteride and 3–40 µg/mL for tadalafil. The methods were validated following ICH guidelines for accuracy, precision, robustness, limit of detection, limit of quantification, and selectivity. The methods were found to be sensitive with LOD values for finasteride and tadalafil of 2.406 µg/mL and 0.876 µg/mL using the second derivative with zero crossing method and 2.229 µg/mL and 0.815 µg/mL using the first derivative of ratio spectra method. The methods were successfully applied for the determination of the studied drugs in their laboratory prepared mixtures, with mean percent recovery for finasteride and tadalafil of 99.37% and 99.17% using the second derivative with zero crossing method and 99.74% and 99.56% using the first derivative of ratio spectra method. Furthermore, the described methods were successfully applied for determination of the studied drugs in Entadfi™ capsules without interference from excipients. Based on the proposed results, the described methods could be utilized as simple method for the quality control of the studied drugs.

## Introduction

Entadfi™ is a prescription combination of finasteride and tadalafil developed by Veru Inc. (Miami, USA) for oral administration. It was recently approved by the FDA in December 2021 for the treatment of urinary tract symptoms caused by benign prostatic hyperplasia in men [1].

Clinically, studies have proven that in men with benign prostatic hyperplasia and prostatic enlargement, coadministration of finasteride and tadalafil results in an early improvement in lower urinary tract symptoms. Additionally, coadministration of finasteride and tadalafil improves erectile function in men who have comorbid erectile dysfunction [2–5].

**Fig. 1:**
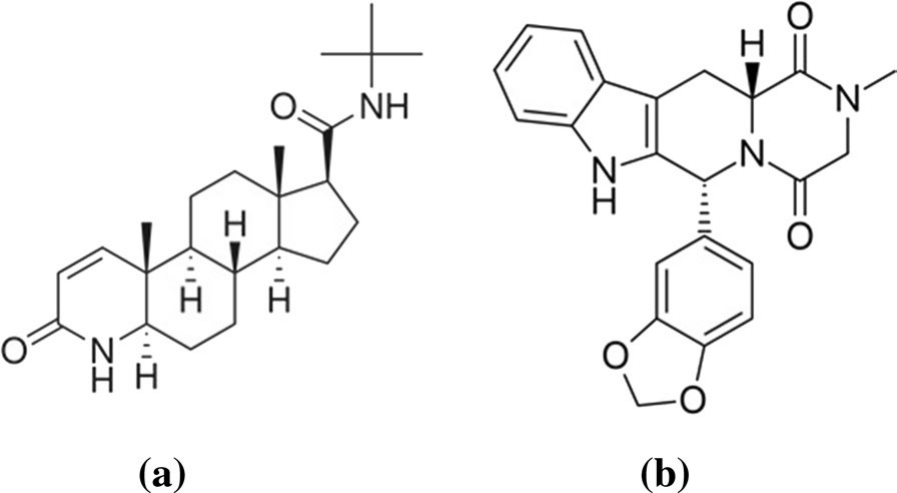
Structural formula of FSD (**a**) and TDL (**b**)

Finasteride (FSD), Fig. 1a, is a specific inhibitor of steroid Type II 5α-reductase, an intracellular enzyme that converts the androgen testosterone into 5α-dihydrotestosterone. It is commonly used to treat benign prostatic hyperplasia, prostate cancer, and androgenetic alopecia. Several analytical approaches have been reported for determination of FSD in pharmaceutical formulations and plasma including HPLC, electrochemical, spectrofluorometric and UV spectrophotometric methods [6–18].

Tadalafil (TDL), Fig. 1b, is a selective inhibitor of cyclic guanosine monophosphate-specific phosphodiesterase type 5. It is indicated for the treatment of erectile dysfunction and the signs and symptoms of benign prostatic hyperplasia. Literature review reveals several reported methods for analysis of TDL in pharmaceutical formulations and plasma such as HPLC, GC, densitometric, electrochemical, spectrofluorometric and spectrophotometric methods [19–30].

Although liquid chromatography-tandem mass spectrometric (LC–MS/MS) method was reported for the simultaneous determination of FSD and TDL [31], the method recommended critical conditions as the presence of internal standard and specific solid phase extraction procedures. Furthermore, LC–MS/MS is a specific instrumental based technique affects the cost and the speed of the quantitative analysis of the compounds of interest. Therefore, authors aimed to develop the simple spectrophotometric methods for simultaneous determination of FSD and TMD in pure and pharmaceutical dosage form. The UV absorption spectra of FSD and TDL show sever overlap, which make the simultaneous determination extremely challenging. Derivative spectrophotometry approach offers a powerful tool for resolving the overlapping spectra and enhancing spectral resolution and selectivity. Selection of the wavelength at which the mixture is analyzed by derivative spectrophotometry is the critical step and may include analysis of the derivative spectra. In the zero-crossing derivative technique, the value of the mixture derivative should, according to the principle of derivative additivity, be equal to the derivative of the second component at the positions where one of the mixture components crosses the zero line. This technique sometimes requires the analysis of the spectrum at many wavelengths. Currently, the ratio spectra derivative method makes selecting a measurement wavelength easy and avoids using the zero-crossing technique to read the derivative result [32, 33].

In this work, second derivative with zero crossing and first derivative of ratio spectra approaches were applied for simultaneous quantification of FSD and TDL in their pure and pharmaceutical formulation.

## Experimental

### Materials and solvent

Pure powders of FSD and TDL were supplied by Pharmakeda Health Company, Cairo, Egypt. Entadfi™ capsules (5 mg FSD and 5 mg TDL per capsule, B. NO.: A1524) were gifted by Pharmakeda Health Company, Cairo, Egypt. HPLC grade methanol was purchased from Sigma-Aldrich, Darmstadt, Germany.

### Apparatus

All measurements were carried out with Shimadzu UV-Visible 1800 Spectrophotometer (Shimadzu Corp., Tokyo, Japan). A 10 mm quartz cuvettes were used to scan the samples absorption spectra. The manipulation of scanned spectra was performed using Shimadzu UV-Probe software version 2.43.

### Standard solutions

FSD and TDL stock solutions of concentration (100 µg/mL) were prepared separately in a 100-mL volumetric flask by dissolving 0.01gm of each drug in methanol.

### Procedures

#### Linearity and calibration graphs

FSD and TDL serial dilutions were made separately by transferring aliquots equivalent to (100–1400 µg) and (30–400 µg) of FSD and TDL, respectively, from their standard solutions (100 µg/mL) into two sets of 10-mL volumetric flasks and diluting to volume with methanol. In the wavelength range of 200–400 nm, the absorption spectra of these dilutions were monitored and recorded against methanol as a blank.

##### Second derivative with zero crossing method (^2^D)

The recorded zero-order absorption spectra of each drug were transformed to its second order derivative using ∆λ = 4 and scaling factor 100. The ^2^D amplitude values were measured at 230.80 and 292 nm for FSD and TDL, respectively. The measured values were plotted against each drug concentrations in µg/mL to get the calibration graphs and the corresponding regression equations were derived.

##### First derivative of ratio spectra method (^1^DD)

The recorded zero-order absorption spectra of each drug were divided by a suitable divisor spectrum from the spectra of the second drug to create the ratio spectra. A spectrum of 25 µg/mL TDL was optimal for FSD ratio spectra, while a spectrum of 80 µg/mL FSD was optimal for TDL ratio spectra. The created ratio spectra of each drug were transformed to its first order derivative using ∆λ = 4 and scaling factor 100. The ^1^DD amplitude values were recorded at 218.80 and 289.60 nm for FSD and TDL, respectively. The recorded values were plotted against each drug concentrations in µg/mL to get the calibration graphs and the corresponding regression equations were derived.

#### Analysis of laboratory mixed solutions

Five samples were made by transferring aliquots of different concentrations from FSD and TDL standard solutions into a set of 10-mL volumetric flasks and diluting to volume with methanol. The samples were analyzed using the procedures outlined under the linearity and calibration graphs for each method, and the concentrations of each drug were calculated.

#### Analysis of pharmaceutical capsules

Contents of 10 Entadfi™ capsules (5 mg FSD and 5 mg TDL per capsule) were weighed and mixed well. A weighed powder equivalent to one capsule was accurately transferred to 100-mL volumetric flask with 50 mL methanol, shaken vigorously for 20 min, filtered, and adjusted to 100 mL with methanol. Further dilution with methanol was made to prepare five samples of different concentrations. The samples were analyzed following the procedure mentioned for each method under the linearity and calibration graphs and the concentration of each drug was computed.

## Results and discussion

### Spectral characteristic

**Fig. 2:**
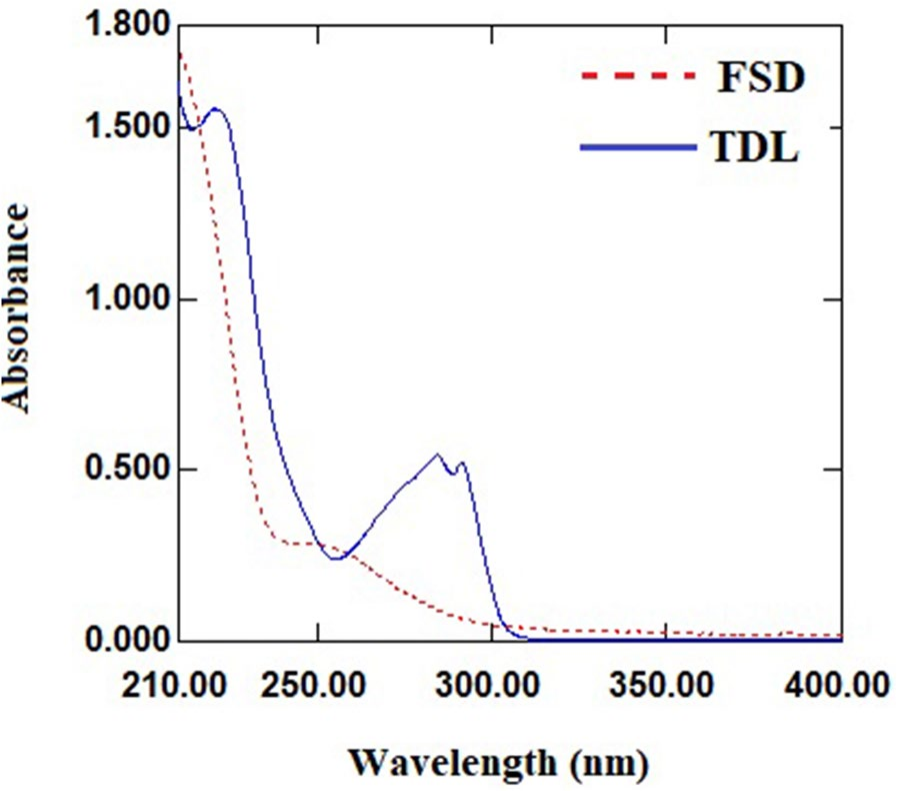
Absorption spectra of FSD (40 µg/mL) and TDL (20 µg/mL)

UV absorption spectra of FSD and TDL show significant overlap, which make the direct simultaneous determination extremely difficult (Fig. 2). Two derivative methods were used to overcome the overlap issue and quantitatively determine FSD and TDL in their mixture. The first approach is the second derivative with zero crossing method, which manipulates the studied drugs’ normal UV spectra, while the second method is the first derivative of ratio spectra method, which manipulates the studied drugs’ ratio spectra.

#### Second derivative with zero crossing method

**Fig. 3:**
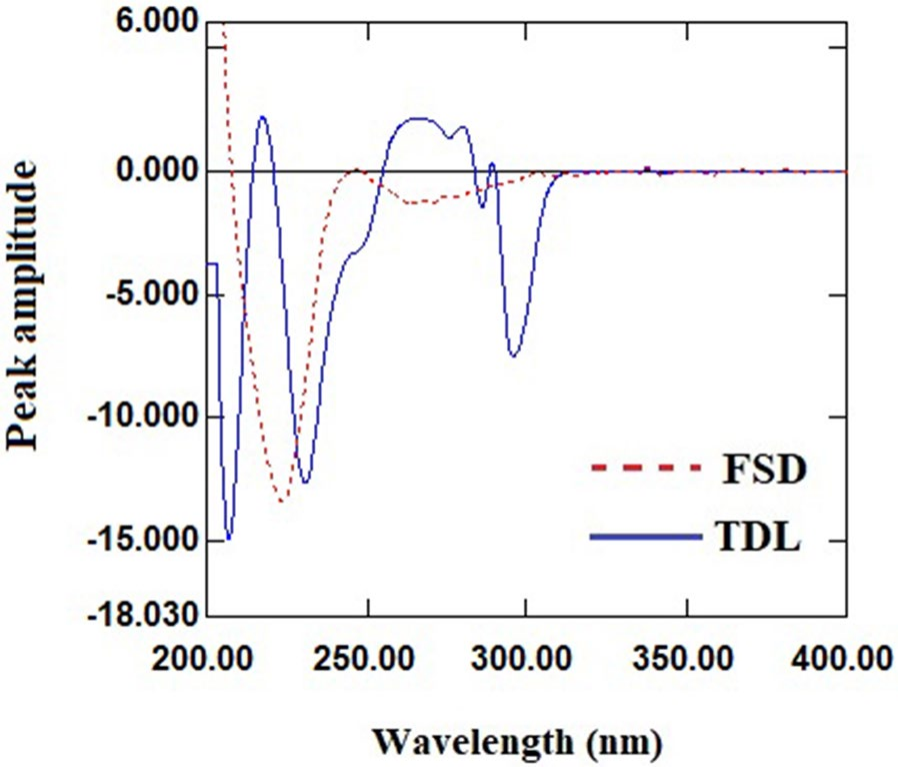
^1^D spectra of FSD (70 µg/mL) and TDL (30 µg/mL)

**Fig. 4:**
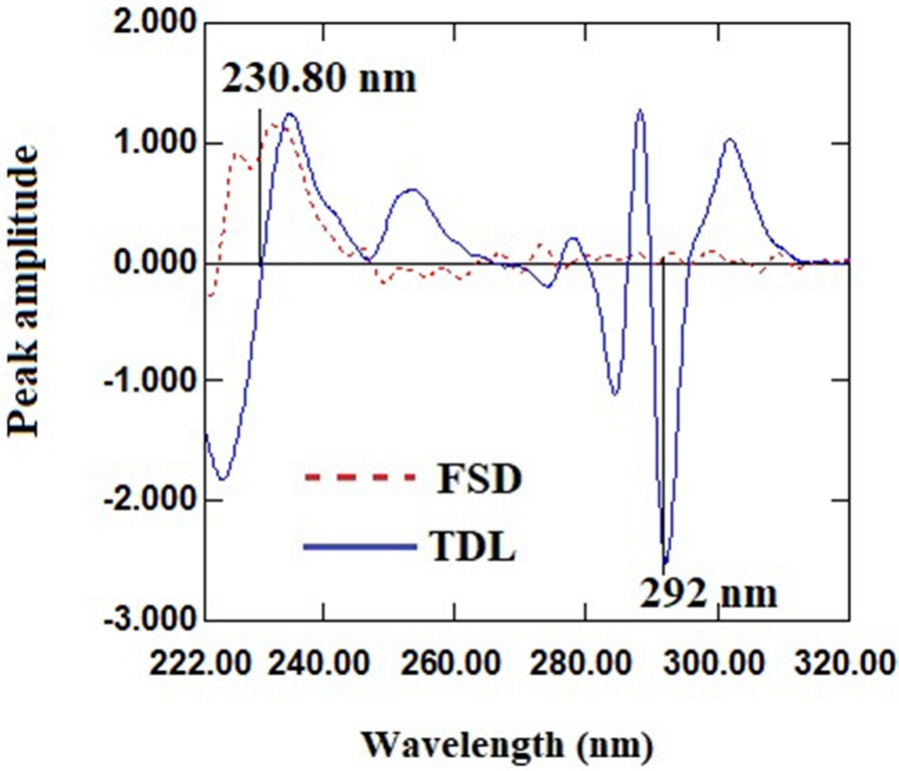
^2^D spectra of FSD (70 µg/mL) and TDL (30 µg/mL)

**Fig. 5:**
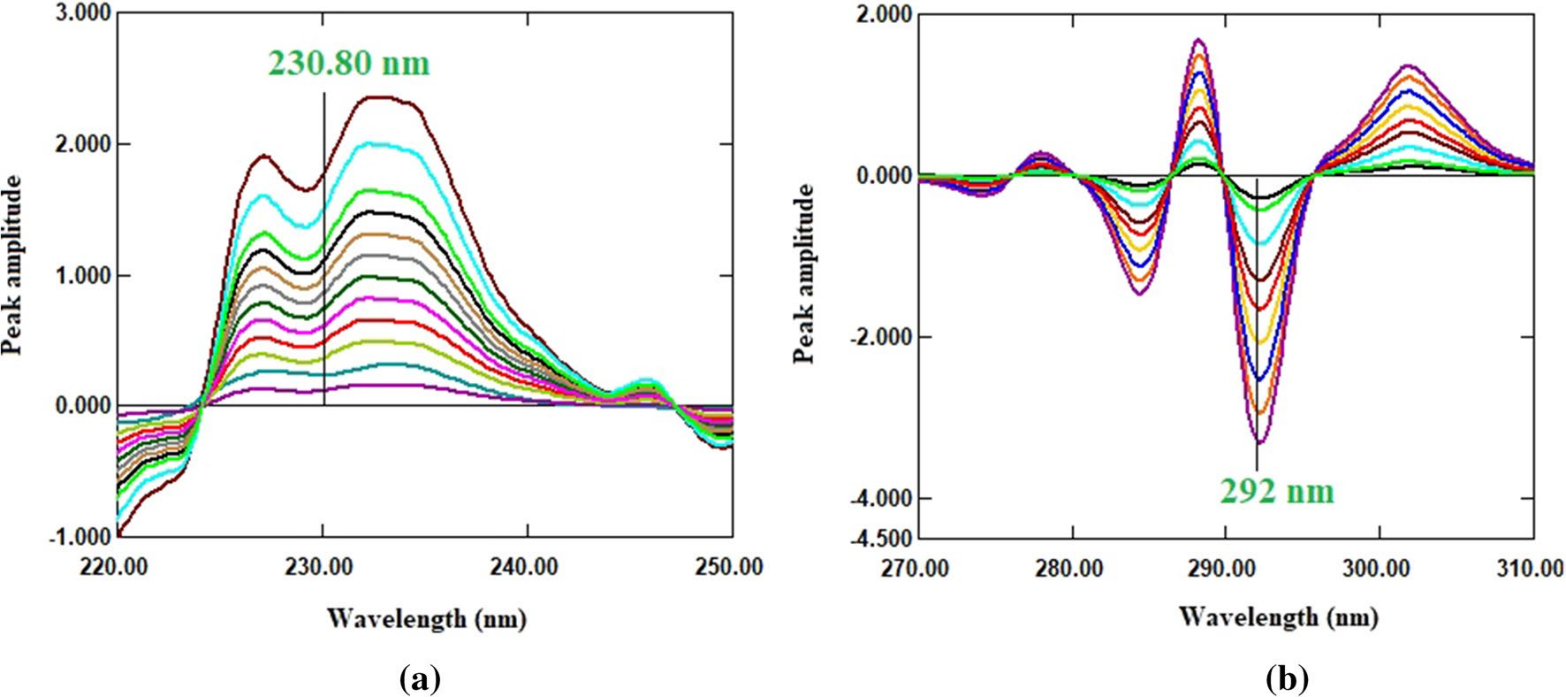
**a** ^2^D spectra of FSD (10–140 µg/mL); **b** ^2^D spectra of TDL (3–40 µg/mL) using

As the first derivative (^1^D) manipulation of the absorbance spectra of FSD and TDL failed to provide feasibility for compounds of interest (Fig. 3), the zero-order absorption spectra of FSD and TDL were converted to the second order derivative using ∆λ = 4 and scaling factor 100. After analyzing the derivative spectra of FSD and TDL to select the wavelength at which each drug was measured in mixture that corresponded to the second drug’s zero-crossing, it was discovered that the wavelengths of 230.80 and 292 nm for FSD and TDL, respectively, provided good linearity and selectivity (Fig. 4). As a result, FSD could be determined selectively in a mixture at 230.80 nm (zero-crossing wavelength of TDL) without interference from TDL (Fig. 5a), and TDL could be determined selectively in a mixture at 292 nm (zero-crossing wavelength of FSD) without interference from FSD (Fig. 5b). The measured ^2^D amplitude values of FSD and TDL at the selected wavelengths were graphed versus each drug concentrations to create the calibration graph, the regression equations were derived, and the concentrations of each drug in mixture were calculated.

#### First derivative of ratio spectra method

**Fig. 6:**
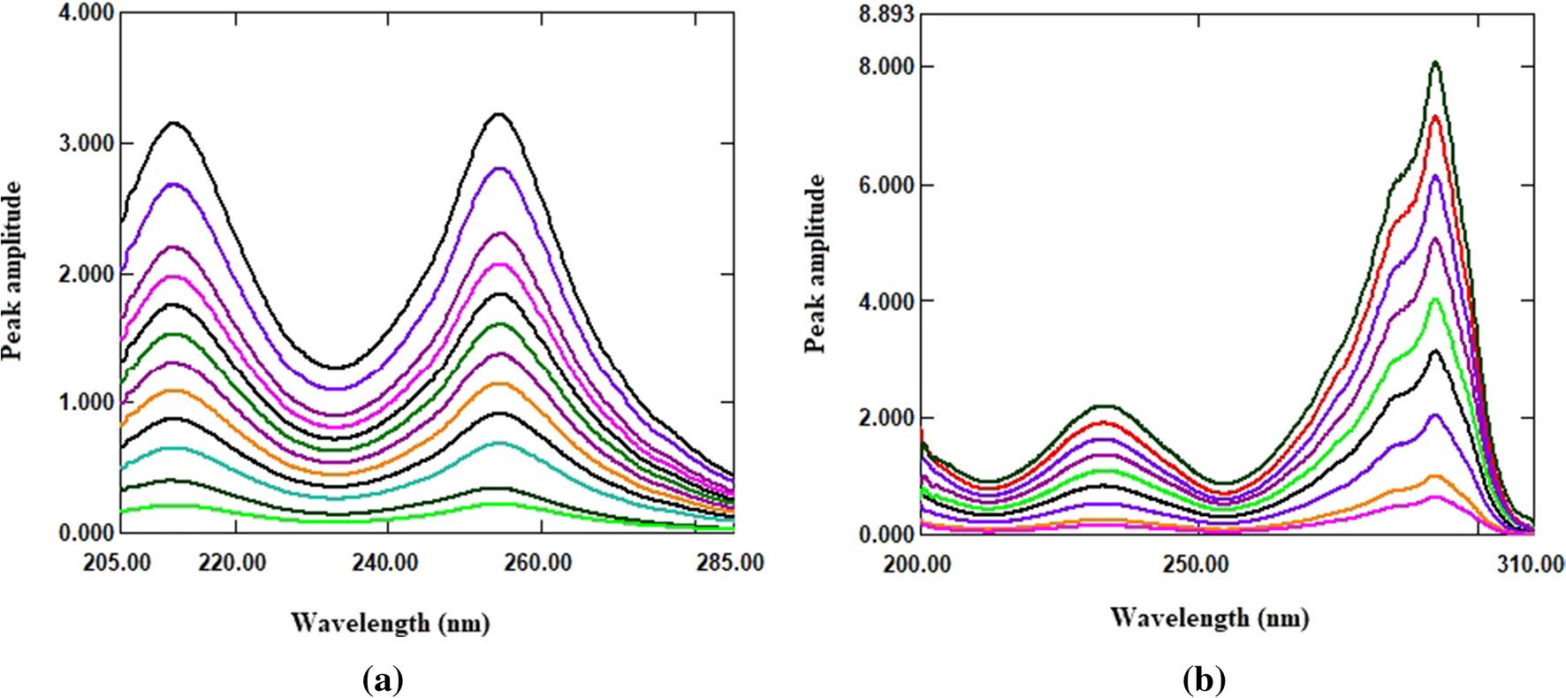
**a** Ratio spectra of FSD (10–140 µg/mL) using 25 µg/mL of TDL as a divisor; **b** ratio spectra of TDL (3–40 µg/mL) using 80 µg/mL of FSD as a divisor

**Fig. 7:**
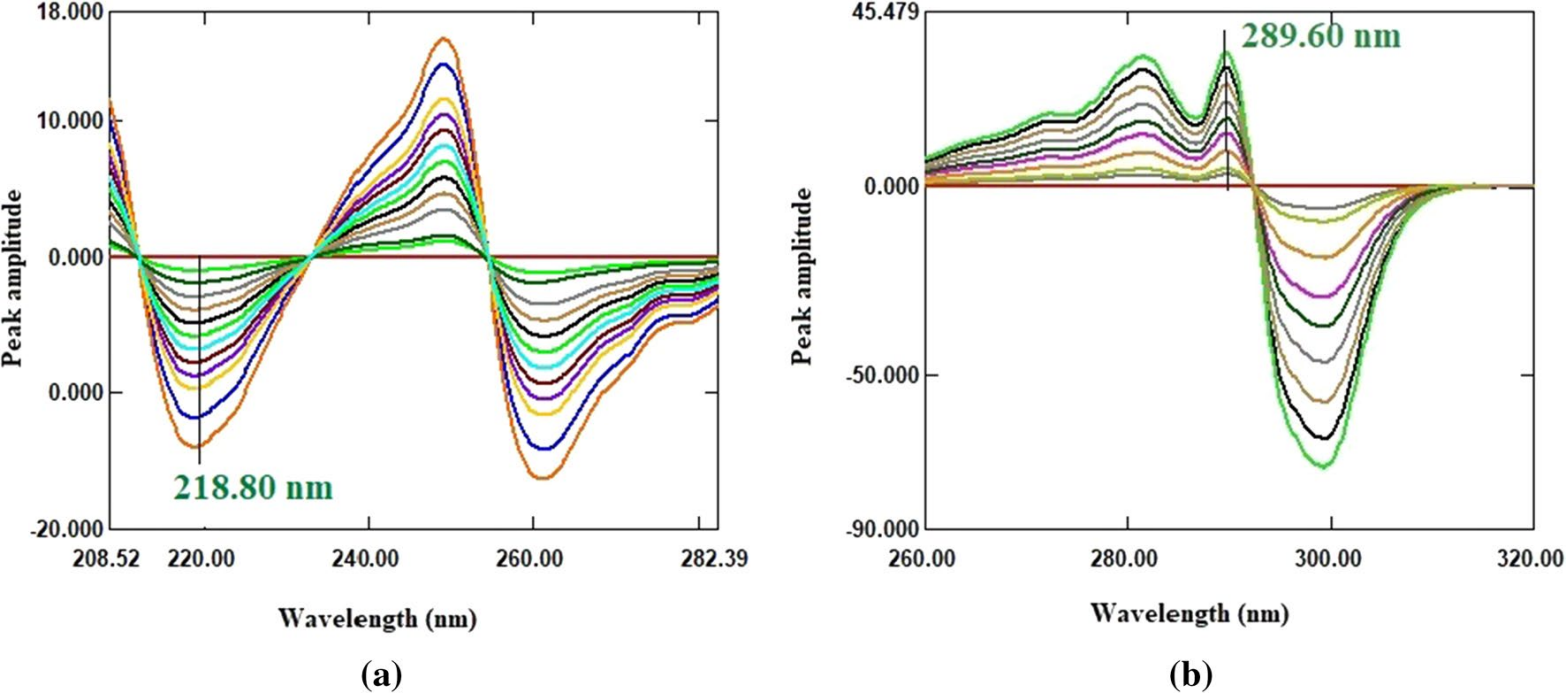
**a** ^1^DD spectra of FSD (10–140 µg/mL) using 25 µg/mL of TDL as a divisor; **b** ^1^DD spectra of TDL (3–40 µg/mL) using 80 µg/mL of FSD as a divisor

The ratio spectra of the studied drugs were derived by dividing each drug’s normal spectra by a suitable spectrum from the spectra of the second drug as a divisor (Fig. 6). Because choosing a divisor is such an important step regarding to signal to noise ratio and sensitivity, various FSD and TDL spectrums were tested as divisors. A spectrum of 25 µg/mL TDL was optimal for FSD ratio spectra, while a spectrum of 80 µg/mL FSD was optimal for TDL ratio spectra. The derived ratio spectra of each drug were transformed to its first order derivative using ∆λ = 4 and scaling factor 100. After examining the linearity and selectivity of the ^1^DD spectra of FSD and TDL at various peak amplitudes, it was revealed that the wavelengths of 218.80 and 289.60 nm for FSD and TDL, respectively, gave good linearity and selectivity. As a result, FSD could be determined selectively in a mixture at 218.80 nm without interference from TDL (Fig. 7a), and TDL could be determined selectively in a mixture at 289.60 nm without interference from FSD (Fig. 7b). The measured ^1^DD amplitude values of FSD and TDL at the selected wavelengths were plotted against each drug concentrations in µg/mL to get the calibration graphs, the regression equations were derived, and the concentrations of each drug in mixture were calculated.

## Method validation

The applied methods were validated according to ICH guidelines [34]. Data listed in Table 1 represents the limits of detection (LOD) and quantitation (LOQ), accuracy, precision, robustness, linearity, and regression parameters. The applied methods demonstrated good linearity in the concentration range of 10–140 µg/mL and 3–40 µg/mL for FSD and TDL, respectively, with acceptable accuracy and precision. The methods were found to be sensitive with LOD values of 2.406 µg/mL and 0.876 µg/mL for FSD and TDL, respectively, in second derivative with zero crossing method and of 2.229 µg/mL and 0.815 µg/mL for FSD and TDL, respectively, in first derivative of ratio spectra method. The described methods exhibited successful application regarding to selectivity for concurrent quantification of FSD and TDL in their laboratory mixed solutions, as shown in Table 2, as well as in Entadfi™ capsules without any interference from each other or from capsule additives which was assured by the recovery data of standard addition technique, as shown in Table 3.

**Table 1:** Regression and validation data for quantitative analysis of FSD and TDL by the proposed methods

Parameters	^2^D		^1^DD	
	FSD	TDL	FSD	TDL
Wavelength (nm)	230.80	292	218.80	289.60
Linearity range (µg/mL)	10–140	3–40	10–140	3–40
Slope	0.0145	0.0822	0.0976	0.8662
Intercept	− 0.0177	0.0223	-0.0574	0.2200
Coefficient of determination (r^2^)	0.9997	0.9996	0.9998	0.9996
LOD (µg/mL)	2.406	0.876	2.229	0.815
LOQ (µg/mL)	7.292	2.654	6.755	2.470
Accuracy (%R)^a^	99.57	99.64	99.52	99.71
Repeatability precision (RSD)^b^	1.192	0.959	0.444	0.650
Intermediate precision (RSD)^b^	1.198	0.963	0.446	0.652
Robustness (%R ± RSD) − Wavelength (± 0.2)	99.78 ± 0.989	100.17 ± 0.731	100.05 ± 0.601	99.56 ± 1.052

^a^Average of 9 determinations (3 concentrations repeated 3 times)

^b^RSD of 9 determinations (3 concentrations repeated 3 times)

**Table 2:** Application of the proposed methods for the determnation of FSD and TDL in their laboratory mixed solutions

Added (µg/mL)		% recovery			
		^2^D		^1^DD	
FSD	TDL	FSD	TDL	FSD	TDL
10	10	99.10	98.14	99.23	99.65
10	20	100.14	99.01	100.35	98.35
20	10	99.90	100.09	100.28	100.21
30	30	98.78	99.99	99.46	100.20
40	40	98.91	98.65	99.40	99.39
Mean ± RSD		99.37 ± 0.615	99.17 ± 0.853	99.74 ± 0.529	99.56 ± 0.766

**Table 3:** Determination of FSD and TDL from Entadfi™ capsules and recovery studies by standard addition method

	^2^D		^1^DD	
	Amount in (µg/mL)	% recovery	Amount in (µg/mL)	% recovery
Capsule 5:5				
FSD	20	99.21	20	100.09
TDL	20	99.13	20	98.59
Recovery of added FSD	10	99.53	10	99.79
	20	98.29	20	99.36
	30	100.04	30	101.01
	Mean ± RSD	99.29 ± 0.906		100.05 ± 0.855
Recovery of added TDL	10	99.05	10	99.62
	20	99.34	20	100.55
	30	98.12	30	100.88
	Mean ± RSD	98.84 ± 0.645		100.35 ± 0.651

## Conclusions

In this paper, we established the first UV spectrophotometric methods for quantification of FSD and TDL in the recently FDA approved Entadfi^™^ capsules. Two UV spectrophotometric procedures, second derivative with zero crossing method and first derivative of ratio spectra method, were found to accurately and precisely estimate FSD and TDL in the pure and in the pharmaceutical formulation. Although the second derivative with zero-crossing method was simpler than the first derivative of the ratio spectra method as it was performed directly on zero-order spectra, the first derivative of the ratio spectra method had greater sensitivity and accuracy.

## Data Availability

All the data associated with this research has been presented in this paper.

## Abbreviations

FDA: Food and Drug Association
FSD: Finasteride
TDL: Tadalafil
^2^D: Second derivative with zero crossing method
^1^DD: First derivative of ratio spectra method
